# Inequities in maternal and child health outcomes and interventions in Ghana

**DOI:** 10.1186/1471-2458-12-252

**Published:** 2012-03-31

**Authors:** Eyob Zere, Joses M Kirigia, Sambe Duale, James Akazili

**Affiliations:** 1 Washington DC, USA; 2 World Health Organization, Regional Office for Africa, Brazzaville, Congo; 3 Tulane University School of Public Health and Tropical Medicine, New Orleans, LA, USA; 4 Navrongo Health Research Centre, Ghana Health Service, Navrongo, Ghana

## Abstract

**Background:**

With the date for achieving the targets of the Millennium Development Goals (MDGs) approaching fast, there is a heightened concern about equity, as inequities hamper progress towards the MDGs. Equity-focused approaches have the potential to accelerate the progress towards achieving the health-related MDGs faster than the current pace in a more cost-effective and sustainable manner. Ghana's rate of progress towards MDGs 4 and 5 related to reducing child and maternal mortality respectively is less than what is required to achieve the targets. The objective of this paper is to examine the equity dimension of child and maternal health outcomes and interventions using Ghana as a case study.

**Methods:**

Data from Ghana Demographic and Health Survey 2008 report is analyzed for inequities in selected maternal and child health outcomes and interventions using population-weighted, regression-based measures: slope index of inequality and relative index of inequality.

**Results:**

No statistically significant inequities are observed in infant and under-five mortality, perinatal mortality, wasting and acute respiratory infection in children. However, stunting, underweight in under-five children, anaemia in children and women, childhood diarrhoea and underweight in women (BMI < 18.5) show inequities that are to the disadvantage of the poorest. The rates significantly decrease among the wealthiest quintile as compared to the poorest. In contrast, overweight (BMI 25-29.9) and obesity (BMI ≥ 30) among women reveals a different trend - there are inequities in favour of the poorest. In other words, in Ghana overweight and obesity increase significantly among women in the wealthiest quintile compared to the poorest. With respect to interventions: treatment of diarrhoea in children, receiving all basic vaccines among children and sleeping under ITN (children and pregnant women) have no wealth-related gradient. Skilled care at birth, deliveries in a health facility (both public and private), caesarean section, use of modern contraceptives and intermittent preventive treatment for malaria during pregnancy all indicate gradients that are in favour of the wealthiest. The poorest use less of these interventions. Not unexpectedly, there is more use of home delivery among women of the poorest quintile.

**Conclusion:**

Significant Inequities are observed in many of the selected child and maternal health outcomes and interventions. Failure to address these inequities vigorously is likely to lead to non-achievement of the MDG targets related to improving child and maternal health (MDGs 4 and 5). The government should therefore give due attention to tackling inequities in health outcomes and use of interventions by implementing equity-enhancing measure both within and outside the health sector in line with the principles of Primary Health Care and the recommendations of the WHO Commission on Social Determinants of Health.

## Background

Barely four years from the target date of 2015 to achieve the Millennium Development Goals (MDGs), there a growing concern on how to accelerate progress to achieving these targets. There is also a heightened concern about equity, as it undermines efforts for sustained improvements across all segments of society and hampers progress towards the MDGs [1,2]. The thrust for a greater focus on equity in human development is gathering momentum at the international level [3]. Equity-focused approach accelerates the progress towards achieving the health MDGs, specially MDGs 4 and 5 related to reducing child and maternal mortality respectively, faster than the current path in a more cost-effective and sustainable manner [4]. The health MDGs referred to in this study, their targets and indicators are presented in Table 1[5].

**Table 1:** Official indicators of Millenium Development Goals on maternal and child health

Millenium Development Goal	Target	Indicator
MDG 4: reduce child mortality	Target 4A: reduce by two-thirds, between 1990 and 2015, the mortality rate in children younger than 5 years	Indicator 4.1: Mortality rate in children younger than 5 years Indicator 4.2: Infant mortality rate Indicator 4.3: Proportion of 1 year-old children immunized against measles
MDG 5: Improve maternal health	Target 5A: reduce by three-quarters, between 1990 and 2015, the maternal mortality ratio Target 5B: Achieve by 2015, universal access to reproductive health	Indicator 5.1: maternal mortality ratio Indicator 5.2: proportion of births attended by skilled health personnel Indicator 5.3: contraceptive prevalence rate Indicator 5.4: Adolescent birth rate Indicator 5.5: antenatal care coverage (at least one visit and at least 4 visits Indicator 5.6: unmet need for family planning

In Africa, modest progress has been registered towards achieving MDGs 4 and 5. However, the rate of progress has been short of what is required to reach the targets. In sub-Saharan Africa, under-five mortality rate decreased from an average of 180 per 1,000 live births in 1990 to 129 per 1,000 live births in 2009 [6]. This translates to an average annual rate of reduction (AARR) of 1.7%, which is far below the AARR of 4.3% required to achieve the MDG 4 target of reducing by two-thirds, between 1990 and 2015, the mortality rate in under-five children. In sub-Saharan Africa, Madagascar, Eritrea and Cape Verde registered under-five mortality AARR of 4.3% or more between 1990 and 2009 [7], and are thus on track to achieve the MDG 4A target. The corresponding AARR for Ghana for the period 1990-2009 was 2.9% [7], and therefore the country is not on track to achieve MDG4A target. Progress in achieving the MDG 5 target of reducing the maternal mortality ratio (MMR) by three-quarters, between 1990 and 2015, has been slow. In sub-Saharan Africa, the maternal mortality ratio decreased from an average of 870 per 100,000 live births in 1990 to 640 per 100,000 in 2008 corresponding to an AARR of 1.7%, which is also far below the required 5.5% to achieve the MDG 5A target of maternal mortality reduction. Although many countries in the region are making progress to achieving the target, only two countries - Equatorial Guinea and Eritrea- are on track. Ghana's AARR in maternal mortality ratio during the same period was 3.3% [8].

Modern health interventions play a significant role in reducing childhood mortality in Africa and other developing countries [9]. There is ample evidence that MDG 4 can be achieved if countries in sub-Saharan Africa and other developing regions of the world target the biggest childhood killers in children - diarrhoea, malaria and pneumonia that account for more than half of under-five deaths. Scaling up of essential curative, preventive and promotive childhood interventions such as immunization, breast feeding, vitamin A supplementation and provision of safe drinking water are necessary to curb childhood morbidity and mortality [10]. Interventions such as focused antenatal care (four visits with a health care provider) and use of skilled attendants during child birth are cost-effective interventions to curb maternal morbidity and mortality.

Despite the modest progress observed, there are substantial inequities in maternal and child health services coverage and health outcomes within and between countries [11]. Current evidence indicates that poor people in both rich and poor countries bear a disproportionately higher burden of ill-health and death, but contrary to expectation have disproportionately less access to health services and interventions than those who are better off [6]. Evidence from various studies in sub-Saharan Africa attests to this [12].

Thus for practical reasons, it is important to examine the equity dimension of health outcomes and interventions in order to better target resources to those who have greater needs and achieve the national and global health targets. This paper, therefore, uses Ghana as case study to assess wealth-related inequalities in maternal and child health outcomes and interventions that are deemed as inequities. Following Whitehead's seminal definition, equity in health is the absence of systematic inequalities in health or in the major social determinants of health among people that have different positions in social hierarchy [13].

### Brief profile of Ghana

Ghana is located on the West Coast of Africa about 750 km north of the equator on the Gulf of Guinea. It has a total land area of 238,305 square kilometers and is bordered on the north by Burkina Faso, on the west by Cote d'Ivoire and on the east by Togo [14]. The country is divided into 10 administrative regions and over 140 districts [15].

Ghana's population was estimated at 24 million in the 2010 Population and Housing Census. The population structure is typical of a developing country with about half of the total population below 15 years of age. Women in Ghana have an average of 4.0 children. The average number of children per woman ranges from 3.1 in urban areas to 4.9 in rural areas. Ghana is a low-income country. The gross national income (GNI) per capita in 2009 was US$ 700 [16].

The burden of disease in Ghana has not changed significantly for decades. Communicable diseases account for about two-thirds of outpatient visits across the nation. Malaria is the main cause of outpatient morbidity. National HIV prevalence increased from 1.7 per cent in 2008 to 1.9 in 2009. The burden of non-communicable diseases such as cardiovascular disorders, diabetes and cancers is emerging as a major challenge to service delivery and a threat to health and national productivity. Similarly, mental health and neurological disorders are also on the increase while trauma and other injuries are significant among outpatients [17].

Maternal mortality continues to be a significant public health challenge despite the increase in antenatal service delivery. Though antenatal care coverage has been sustained at a high level of about 85%, deliveries by skilled personnel have declined from 44.5% in 2006 to 34.9% in 2007. Maternal mortality ratio has increased from 187.2/100,000 to 229.9/100,000 live births respectively.

Ghana Health Service is organized at three main levels, national, regional and district. Payment mechanism for health care is a combination of health insurance and out-of-pocket payment.

## Methods

### Data sources

Data is extracted from Ghana demographic and health survey (GDHS) of 2008 report. The 2008 DHS was a nationally representative survey of 11,778 households comprising 4,916 women in the age group 15 to 49 years and 4,568 men aged 15-59 years. The survey employed a two-stage sampling based on the 2000 Population and Housing Census [18].

### Variables and definitions

#### Maternal and child health outcomes

The health outcomes included in this study are defined in GDHS 2008 as indicated in Table 2[18].

**Table 2:** Maternal and child health outcomes included in the study and their definitions

Health outcome	Definition/measurement
Infant mortality rate (IMR)	Probability of dying between birth and exactly age 1
Under-five mortality rate (U5MR)	Probability of dying between birth and exact age five
Perinatal mortality rate	Includes pregnancy losses of at least seven months gestation (stillbirths) and deaths among live births that occurred within the first seven days of life (early neonatal deaths)
Stunting	Height-for-age of under-five children below minus two standard deviations of the WHO Child Growth Standards median.
Underweight	Weight-for-age of under-five children below minus two standard deviations of the WHO Child Growth Standards median.
Wasting	Weight-for-height of under-five children below minus two standard deviations of the WHO Child Growth Standards median.
Anaemia in children 6-59 years	Haemoglobin concentration below 11 g/dL
Acute respiratory infection (ARI) in children	Cough accompanied by short, rapid breathing in the two weeks preceding the survey
Diarrhoea in children	Mothers asked whether any of their children under five years of age had diarrhoea during the two weeks preceding the survey
Nutritional status of women age 15-49 years	Defined as weight in Kilograms divided by height squared in metres (Kg/m^2) ^(Body Mass Index - BMI). A BMI of < 18.5 was regarded as *thin*, 18.5-24.9 *normal*, 25-29.9 *overweight*, and ≥ 30 *obese*.
Anaemia in women age 15-49 years	Haemoglobin concentration below 11 g/dL in pregnant women and below 19 g/dL in non-pregnant women

#### Maternal and child health interventions

The interventions included in this study are defined in GDHS 2008 as indicated in Table 3[18].

**Table 3:** Maternal and child health interventions included in the study and their definitions

Intervention	Definition/measurement
Child immunization	A child is considered fully vaccinated when he/she gets one dose each of BCG and measles, three doses each of polio vaccine and DPT
Treatment of diarrhoea in children	Percentage of children under-five with diarrhoea in the two weeks preceding the survey for whom advice or treatment was sought from a health facility or provider
Treatment of fever in children	Percentage of children under-five with fever in the two weeks preceding the survey for whom advice or treatment was sought from a health facility or provider
Skilled birth attendance	Percentage of births delivered by skilled providers that include doctor, nurse, midwife, auxiliary midwife and community health officer
Delivery at health facility	Percentage of births delivered in public and private sector health facilities
Delivery at public facility	Percentage of births delivered in public sector health facilities
Home delivery	Percentage of births delivered at home
Current use of modern contraceptive method	Percentage of currently married women age 15-49 who use modern contraceptive methods that include female sterilization, temporary female methods (pill, IUD, injectable, implants, female condom, diaphragm, foam/jelly and lactational amenorrhoea method) and male condom
Caesarean section	Percentage of live births in the five years preceding the survey delivered by Caesarean section
ITN use, child	Percentage of children in all households who slept under ITN the past night
ITN use, pregnant woman	Percentage of pregnant women age 15-49 who slept under ITN past night
Intermittent preventive treatment, pregnant woman	Percentage of women age 15-49 years who had a live birth in the two years preceding the survey who received at least 2 doses of sulphadoxine-pyrimethamine (SP), at least one during antenatal care visit

#### Analytical methods

##### Measurement of inequities

The measurement of inequities in maternal and child health outcomes and access to health care interventions entails three steps [19]: (i) identification of the health outcome or intervention whose distribution is to be measured; (ii) classification of the population into different strata by a selected equity stratifier; and (iii) measuring the degree of inequality.

The variables of interest, that is the maternal and child health outcomes and interventions are listed in Tables 2 and 3. In the Demographic and Health Surveys, the socio-economic stratifier used is household wealth, which is derived from the household ownership of assets such as television, car etc. and dwelling characteristics such as flooring material and source of drinking water. In this study, we have used wealth quintiles that are provided in the DHS report. In this study, we have used wealth quintiles that are provided in the DHS report. Each asset was assigned a weight (factor score) generated through principal components analysis, and the resulting asset scores were standardised in relation to a normal distribution with a mean of zero and standard deviation of one. Each household was then assigned a score for each asset, and the scores were summed for each household; individuals were ranked according to the total score of the household in which they resided. The sample was then divided into quintiles from one (lowest) to five (highest). A single asset index was developed for the whole sample; separate indices were not prepared for the urban and rural populations [18].

To date, various measures have been used in the measurement of inequities in health and health care. Of the available measures only the slope index of inequality (SII), the relative index of inequality (RII) and the concentration index have the following desirable characteristics: (i) they reflect the socio-economic dimension of health inequalities; (ii) they reflect the experience of the entire population rather than only two groups such as wealth quintiles one and five and (iii) they are sensitive to changes in the distribution of the population across socio-economic groups [20].

In this study, the presence or absence of inequities is measured using population-weighted, regression-based measures: SII and RII. These measures are selected for this analysis because of their ease of interpretation. The SII and RII are based on the socio-economic dimension to inequalities in health and are weighted by the social group proportions [20,21]. The SII is a measure of absolute effect, while the RII measures relative effect. The SII and RII are interpreted as the effect on health or utilization of health care intervention of moving from the lowest to the highest socio-economic group, which is from wealth quintile 1 to wealth quintile 5.

To compute the SII, social groups (wealth quintiles) are ranked from lowest to highest. The population in each wealth quintile covers a range in the distribution of the population and is given a score based on the midpoint of its range in the cumulative distribution in the population. The SII is the linear regression coefficient (slope of the regression line) showing the relationship between a group's (wealth quintile in this case) health and its relative socio-economic rank. In other words:

(1) $$y_{i}=\beta_{0}+\beta_{1}x_{i}+\varepsilon$$

Where:

*y_i _*is the value of the health variable of wealth quintile *i;*

*x_i _*is the relative rank of wealth quintile *i;*

*β*_0 _is the constant or intercept term, which captures the value of *y *when *x *equals zero;

*β_i _*is the slope coefficient (or parameter), and it indicates the amount the y will change when x changes by one unit; and

*ε *is the stochastic error (or disturbance) term that captures the variation in *y *that cannot be explained by the included *x_i_*.

The coefficient *β_1_*represents the SII. The relative index of inequality is derived from the SII as follows:

(2) $$RII=\frac{SII}{\mu }=\frac{\beta_{1}}{\mu }$$

where, *μ *is the population average of the specific health variable.

However, because we are making use of grouped data, the error term of the regression equation is heteroskedastic making the Ordinary Least Squares (OLS) estimates inefficient. To avoid this problem, the SII is therefore estimated using Weighted Least Squares (WLS) [20]. This can be done by running OLS regression on the following transformed equation:

(3) $$y_{i}\sqrt{n_{i}}=\beta_{0}\sqrt{n_{i}}+\beta_{1}x_{i}\sqrt{n_{i}}+\varepsilon_{i}$$

Where, *n_i_*is the size of wealth quintile "*i*", that is the number of individuals in each wealth quintile. It has to be noted that there is no constant term in Equation (3).

SII and RII avoid the defects of the range measures such as rate difference between the wealthiest and poorest quintiles or rate ratio of these two extreme quintiles. They reflect the experience of the entire population as opposed to extreme groups such as wealth quintiles 1 and 5 and are sensitive to the distribution of the population across socio-economic groups (wealth quintiles). The disadvantage of the SII/RII is that it can only be applied to socio-economic variables that can be ordered hierarchically. Besides, linearity is assumed in the regression model; non-linearity would lead to bias in the magnitude of the index.

Data was analyzed using STATA 10 statistical software.

## Results

### Descriptive statistics

Table 4 depicts the values of the selected maternal and child health outcome indicators according to the GDHS 2008. Distribution of all indicators in this study by wealth quintile is provided in Additional file 1: Annex 1.

**Table 4:** Ghana selected maternal and child health indicators

Indicator	Value
IMR per 1000 live births	50
U5MR per 1000 live births	80
Perinatal mortality rate per 1000 live births	39
Stunting (%)	37.8
Underweight (%)	17
Wasting (%)	10.7
Anaemia among children 6-59 months (%)	77.9
Acute respiratory infection (%)	5.5
Diarrhoea (%)	19.8
Body mass index < 18.5 (thin) (%)	8.6
Body mass index, 25-29.9 (overweight) (%)	20.7
Body mass index ≥ 30 (obese) (%)	9.3
Anaemia among women age 15-49 years (%)	58.7

The infant and under-five mortality rates are relatively better when compared to averages of sub-Saharan Africa, which in 2009 were 81 per 1,000 live births and 129 per 1,000 live births respectively. However, they still remain high. The level of childhood malnutrition is very high and of public health significance. According to the World Health Organization's prevalence cut-off values for public health significance [22], the level of malnutrition in Ghana is classified as very high prevalence of stunting and high prevalence of underweight and wasting. It is also noted that the prevalence of overweight and obesity combined is more than three times the prevalence of thinness (or underweight) among women in the 15-49 years age group. It is also observed that more than a third of the Ghanaian women are thin, overweight or underweight, implying that only about 60% have a normal body weight. The table further indicates that the prevalence of anaemia among children and women is alarming and a public health problem. Almost 4 out of 5 children 6-59 months of age and more than half of women in the 15-49 years age group were found to be anaemic (Table 4).

**Figure 1:**
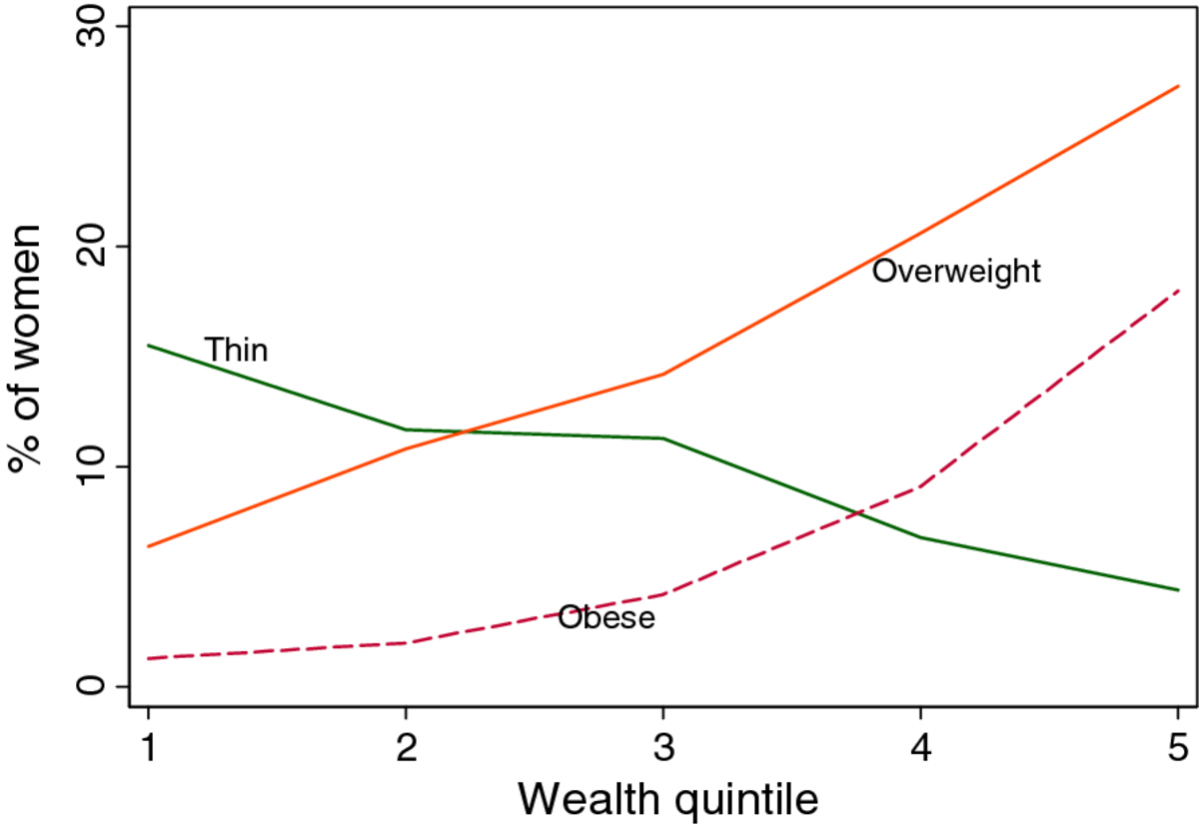
**Malnutrition among women age 15-49 years in Ghana**.

Analysis of the BMI by household wealth quintile gives a mixed picture on the prevalence of malnutrition among women (Figure 1). The prevalence of underweight (thin) is high among the poorest quintiles and decreases with the improvement of the socio-economic status of the household. However, underweight and obesity are more prevalent among women in the top wealth quintile.

Table 5 depicts the values of selected maternal and child health intervention indicators according to the GDHS 2008. Coverage of vital maternal and child health services is critically low except for childhood immunization and caesarean delivery.

**Table 5:** Ghana selected maternal and child health intervention indicators

Indicator	Value
Children 12-23 months age who received all basic vaccinations (%)	79
Children under-five who slept under an ITN (%)	28.2
Children under-five with diarrhoea for whom advice or treatment was sought from a health facility or provider (%)	41
Births assisted by a skilled provider (%)	58.7
Delivery in a health facility (%)	57.1
Delivery in a public sector health facility	48.4
Delivery in a private sector health facility	8.7
Home delivery (%)	42
Delivery by Caesarean section	6.9
Currently married women age 15-49 who use modern contraceptive methods (%)	16.6
Pregnant women who slept under an ITN (%)	19.9
Intermittent preventive treatment (IPT) among women during pregnancy (%)	43.7

**Figure 2:**
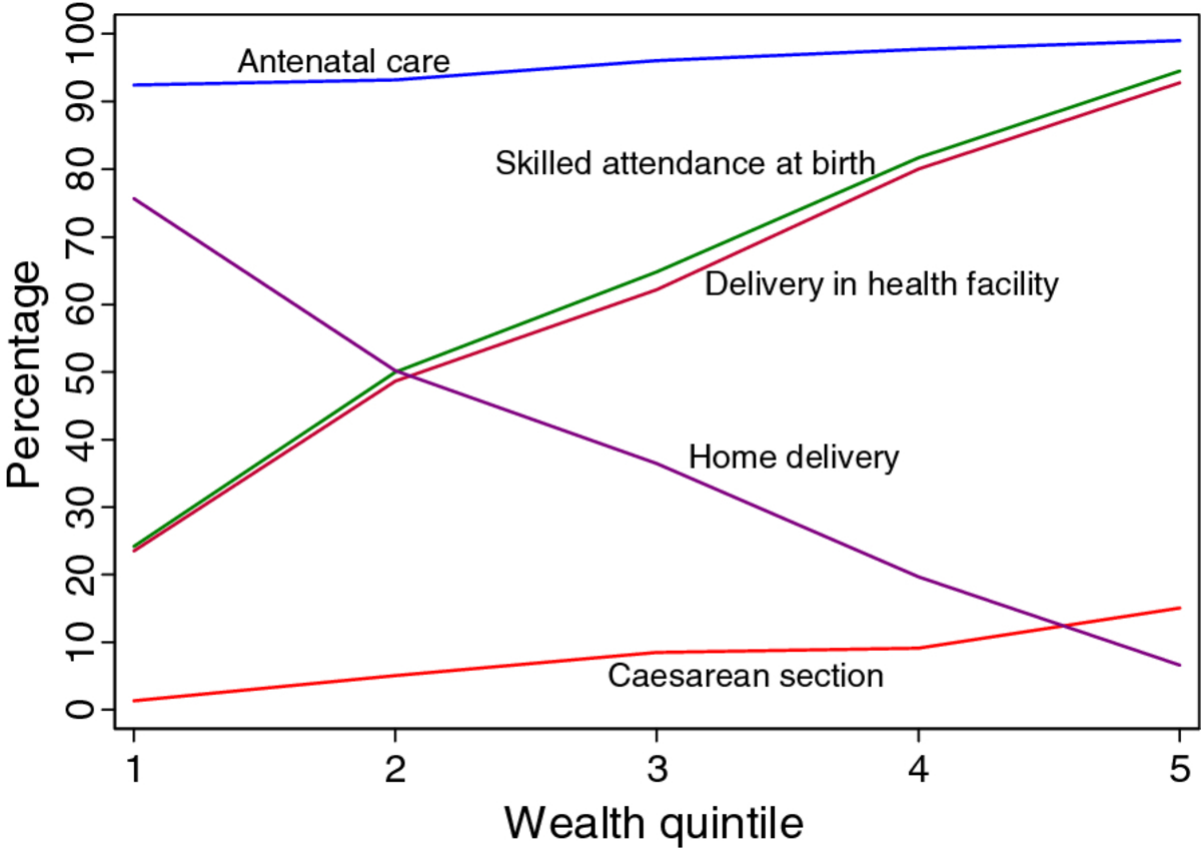
**Coverage rates of selected maternal health interventions by household wealth quintile**.

The coverage rates depicted in Table 5 would give a comprehensive view of the situation if the distributional dimension is included. As an example, the case of maternal health interventions is depicted in Figure 2. The figure indicates that there is no difference in the use of antenatal services among pregnant women from different socio-economic backgrounds. However, all the other indicators favour women from the richer wealth quintiles. Home delivery is mainly practiced by poor women and declines sharply with improvements in the economic status of the woman. The population average rate of Caesarean delivery is about 6.9%; disaggregation of the rate by wealth quintile shows that it is only 1.3% among the bottom 20% compared to 15% among the wealthiest 20%.

### Inequities in maternal and child health outcomes

Inequities in maternal and child health outcomes exist when there are inequalities in each of the selected indicators that are systematically related to household wealth derived from asset indices. Table 6 presents the SII and RII for the health outcomes.

**Table 6:** Slope and relative indices of inequality for selected maternal and child health outcomes

Indicator		95% CI^#^			95% CI	
	SII	Lower	Upper	RII	Lower	Upper
IMR	-11.9	-71.9	48.2	0.24	-1.44	0.96
U5MR	-46.5	-117	24.1	-0.58	-1.46	0.30
Perinatal mortality rate	17.2	-52.4	86.8	0.44	-1.34	2.23
Stunting	-25.3*	-39.5	-11.1	-0.90	-1.04	-0.29
Underweight	-15.4*	-22.5	-8.3	-1.12	-1.32	-0.49
Wasting	-4.6	-10.9	1.8	-0.54	-1.28	0.21
Anaemia in children	-32*	-52.7	-11.3	-0.41	-0.68	-0.14
ARI in children	-1.8	-10.2	6.7	-0.33	-1.85	1.23
Diarrhoea in children	-16.4*	-27.3	-5.6	-0.82	-1.38	-0.28
BMI < 18.5 (thin)	-12.2*	-21.9	-2.5	-1.42	-2.55	-0.29
BMI 25-29.9 (overweight)	24.5*	5.2	44.6	1.18	0.25	2.15
BMI ≥ 30 (obese)	23.0*	10.7	35.3	2.47	1.15	3.38
Anaemia in women	-9.9*	-18.4	-1.3	-0.17	-0.31	-0.02

^# ^confidence interval; * *P < 0.05*

From Table 6 it is observed that the SII and RII for the indicators IMR, U5MR, perinatal mortality rate, wasting and ARI in children are not statistically significant implying that there are no inequities in these health outcomes. The prevalence of Stunting significantly decreases by 25 percentage points as we move from wealth quintile 1 to 5. In relative measures this implies that as one moves from the bottom wealth quintile to the top, the prevalence of stunting decreases by 90% (RII = -0.90). Similarly, anaemia in children and women, childhood diarrhoea, and underweight (thinness) in women 15-49 years of age demonstrate inequities favouring the wealthiest, as the respective rates decrease significantly among those in the top wealth quintile. A point worth of note is that, while underweight in women decreases by 142% (RII = -1.42) in women in the wealthiest quintile as compared with those in the bottom wealth quintile, overweight and obesity demonstrate a different trend. Overweight and obesity increase by 118% (RII = 1.18) and 247% (RII = 2.47) respectively as we move from the bottom quintile to the top. The prevalence rates of overweight and obesity are significantly higher among those who are in a better economic position.

### Inequities in maternal and child health interventions

The analysis shows that treatment of diarrhoea in children, children receiving all basic vaccines and sleeping under ITN (both children and pregnant women) have no wealth-related gradient.

Table 7 presents the SII and RII values of the selected interventions. Skilled attendance at birth, which is an important intervention for the achievement of the MDG 5 target of curbing maternal mortality, is observed to increase by 150% among women in the highest wealth quintile as compared to those in the lower. Similarly deliveries in public or private sector health facility increase among women in the highest socio-economic group by the same magnitude. In contrast, home delivery decreases by about 200% when we move from those in the lowest wealth quintile to those in the top. Thus, home delivery is practiced more by the poorest. The rate of caesarean section and the use of modern contraceptive methods increase by more than 200% and 72% respectively among the wealthiest women. Furthermore intermittent preventive treatment of malaria during pregnancy manifested an increase by 55% among those in the wealthiest quintile. Use of ITNs both among children and pregnant women did not show any socio-economic inequalities.

**Table 7:** Slope and relative indices of inequality for selected maternal and child health interventions

Indicator		95% CI			95% CI	
	SII	Lower	Upper	RII	Lower	Upper
Treatment of diarrhoea in children	3.6	-11.0	18.3	0.09	-0.27	0.45
Received all basic vaccines - children	13.9	-3.9	31.7	0.18	-0.05	0.40
Skilled attendance at birth	87.5*	75.6	99.5	1.5	1.29	1.70
Delivery in health facility	86.1*	74.2	97.9	1.5	1.30	1.71
Delivery in public sector health facility	65.4*	47.4	83.3	1.4	0.98	1.72
Delivery in private sector health facility	20.6*	7.3	33.8	2.4	0.84	3.89
Home delivery	-85.5*	-97.8	-73.2	-2.0	-2.33	-1.47
Caesarean section	15.2*	8.7	21.8	2.20	1.26	3.16
Use of modern contraceptive methods	11.9*	11.3	16.5	0.72	0.68	0.99
Child slept under ITN	-3.1	-12.3	6.1	-0.11	-0.43	0.22
Pregnant woman slept under ITN	-16.2	-45	12.1	-0.81	-2.26	0.61
IPT during pregnancy	23.9*	1.5	46.4	0.55	0.03	1.06

*P < 0.05

## Discussion and conclusion

This study attempts to examine socio-economic inequalities in maternal and child health outcomes and interventions in Ghana using population weighted, regression-based measures of slope and relative index of inequality. Assessing these socio-economic inequalities, which in this case are referred to us inequities, is very important for evidence-based decision-making and targeting scarce public resources to those with more need. Achieving the relevant health-related MDG targets becomes difficult in the presence of inequities in health and health care that disadvantage the poor, since it is among the poorest groups that the MDG indicators are not good and there is a significant potential for improvement in these groups [2].

The selected maternal and child health outcomes indicate that a challenging task lies ahead to improve the health status of women and children in Ghana, although some of the indicators appear relatively better compared to average figures for countries in sub-Saharan Africa. The high rates of childhood mortality and malnutrition among children and women are of great concern if the country is to accelerate progress towards achieving the MDGs related to maternal and child health. Anaemia is a severe public health problem in Ghana, as it exceeds the 40% cut-off mark for the classification of public health significance of anaemia in populations [23].

The overall coverage levels of the selected maternal and child health interventions are still low with the exception of immunization coverage and Caesarean delivery. It should, however, be noted that these average figures mask the reality. For example, while the population average rate of Caesarean delivery is about 6.9%; disaggregation of the rate by wealth quintile shows that the rate among the wealthiest 20% is 14 times more than the rate among the poorest 20% (15% vs. 1.3%). Although there is a debate, a population-based Caesarean section rate of 5-15% has been considered as the acceptable level to ensure the best outcomes for mothers and children [24]. The proportion of deliveries by Caesarean section in a geographical area is a measure of access to and use of obstetric emergency care for averting maternal and neonatal deaths [19]. Therefore, there is under-provision of Caesarean section to the poorest segment of society, which poses a serious challenge to curbing maternal mortality. This impedes the achievement of MDG 5.

The slope and relative indices of inequality reveal the existence of statistically significant gradients in the following health outcome measures: stunting, underweight, anaemia and diarrhoea in under-five children; and, underweight/thin (BMI < 18.5), overweight (BMI = 25-29.9), obese (BMI ≥ 30) and anaemia in women in the age group 15-49 years. With the exception of overweight and obesity in women 15-49 age, all other indicators show a pro-wealthy inequity. This implies that the rates of these health outcome indicators decline significantly as one moves from the poorest wealth quintile to the wealthiest quintile. In contrast, the childhood mortality indicators - IMR, U5MR and perinatal mortality rate - and wasting in under-five children do not exhibit wealth related gradients that may be labeled as inequities.

The nutritional status of under-five children is one of the indicators of household well-being and determinants of child survival [25]. The world Health Organization recommends it as one of the measures of health status to assess equity in health [26]. Besides being an important cause of under-five mortality [27,28], childhood malnutrition may adversely affect a child's intellectual development and consequently, health and productivity in later life [29,30]. Wealth-related inequities in stunting (chronic malnutrition) and underweight in favour of the top wealth quintile clearly demonstrate the well-established link with socio-economic deprivation [31]. Hence, addressing inequities in stunting and underweight will entail initiating and implementing a multi-sectoral action and tackling the broader social determinants of malnutrition in line with the recommendations of the WHO Commission on Social Determinants of Health [32].

The overall prevalence of anaemia among under-five children is consistent of settings where malaria is endemic [33]. Anaemia affects the poorest of society disproportionately [22]. This is attested to by the finding of this study of the existence of inequities in anaemia prevalence in favour of children from wealthier segment of society. This inequity will adversely affect progress towards MDG 4, as anaemia is associated with an increased risk of child mortality [22].

The wealth-related gradient in childhood diarrhoea that is to the disadvantage of children from the poorest wealth quintile is not surprising. diarrhoea is the second main cause of death among children under-five globally [34]. It is therefore a priority to control diarrhoea in children in Ghana in order to accelerate progress towards the MDG 4 target.

Inequities in health outcomes (including diarrhoea) that are to the disadvantage of the poorest children result from increase exposure to disease risk factors; low coverage of preventive interventions and limited access to curative services [12,35]. These problems require interventions both within and outside the health sector that the stewards of health in Ghana have to address simultaneously in order to expedite progress towards the MDGs in a sustainable manner.

The BMI indicator suggests the co-existence of overweight and obesity on the one hand and underweight on the other among women 15-49 years of age. While there are inequities in favour of the rich in the prevalence of underweight (thin), overweight and obesity manifest inequities in the opposite direction - to the advantage of the poor. Underweight significantly decreases in the wealthiest quintile of the population compared to those in the bottom 20%. However, overweight and obesity increase in the wealthiest quintile compared to the poorest 20%. Ghana, like other developing countries may be experiencing the double burden of malnutrition. Abnormal BMI has an adverse effect on pregnancy outcomes [36] and is likely to impede progress towards achieving the MDGs on maternal and child health. It is therefore essential to put appropriate measures that help women to maintain normal BMI.

Anaemia among women likewise manifests pro-wealthy inequities. However, it should be noted that even among the wealthiest quintile, the rate is in the range that is labeled as severe public health problem. Anaemia poses an increased risk for maternal and child mortality [22] and is likely to directly thwart efforts to achieving the MDGs 4 and 5 targets. Although the poorest have to be targeted with preventive and curative interventions, given the magnitude of the problem, it is vital to also implement measures aimed at universal coverage with interventions against anaemia.

The results indicate that the following interventions do not manifest wealth-related gradients: treatment of diarrhoea in children, childhood vaccines, sleeping under ITN (child and pregnant woman). Skilled attendance at birth, place of delivery (health facility, public health facility, private health facility) and Caesarean delivery increase significantly among the wealthiest compared to the poor. It is interesting to note that even the publicly-funded child delivery services are used more by the rich than the poor, reinforcing the assertion that government health spending in Africa benefits the richest of society more than the poorest [37]. It is evident that access of the poor to emergency obstetric care services has to be increased in order to improve maternal health conditions. However, this should not only be limited to increase in the supply of emergency obstetric care. Demand side factors (e.g. individual, household and community level characteristics) should also be examined in order to address any obstacles to utilizing these services by the poorest women.

Not unexpectedly, home delivery significantly decreases as we move from the poorest wealth quintile to the highest. There is an urgent need to reverse this situation so that more women from the poorest of society will give birth at health facilities under the supervision of skilled birth attendants. This will go a long way in bridging inequities and accelerating the progress towards achieving the maternal mortality reduction target of MDG 5.

The fact that intermittent preventive treatment for malaria during pregnancy has a pro-rich inequity may possibly raise a question about the responsiveness of the health system. For example, the Ghana DHS 2008 shows that while 80% of women in the wealthiest quintile are informed of signs of complications of pregnancy, only 55% of those in the poorest quintile are provided with the same information. Thus, socio-economic status seems to affect the quality of care provided to pregnant women.

In summary, pro-rich inequities in most of the maternal and child health interventions in Ghana are wide spread and need to be addressed vigorously in order to improve the health conditions of the poorest women and children and expedite progress towards achieving the MDGs related to maternal and child health in the few years left to the target date of the MDGs.

## Competing interests

The authors declare that they have no competing interests.

## Authors' contributions

EZ designed the study, performed the analysis and drafted the report. JMK, SD and JA contributed to the write up and revision of the manuscript. All authors read and approved the final manuscript.

## Pre-publication history

The pre-publication history for this paper can be accessed here:

http://www.biomedcentral.com/1471-2458/12/252/prepub

## Supplementary Material

Additional file 1 **Annex 1. Distribution of indicators by wealth quintile**. Click here for file
