# Supplementary material for: Inequities in maternal and child health outcomes and interventions in Ghana
Source: BMC Public Health. 2012 Mar 31;12:252. doi: 10.1186/1471-2458-12-252 (PMC3338377; doi:10.1186/1471-2458-12-252)
Supplement: Additional file 1 — Annex 1. Distribution of indicators by wealth quintile. [file 1471-2458-12-252-S1.DOCX]

| Indicator | Wealth inequality | | | | |
| --- | --- | --- | --- | --- | --- |
|  | Q1- poorest  (number) | Q2  (number) | Q3 (number)  (number) | Q4  (number) | Q5-richest  (number) |
| Infant mortality rate (per 1000 LB) | 59  (727) | 45  (629) | 70  (525) | 45  (548) | 46  (406) |
| Under-five mortality rate (per 1000 LB) | 103  (727) | 79  (629) | 102  (525) | 68  (548) | 60  (406) |
| Perinatal mortality rate per 1000 LB) | 33  (752) | 27  (646) | 61  (559) | 36  (568) | 44  (425) |
| Stunting (%) | 35.1  (623) | 34.1  (573) | 28.3  (468) | 21.4  (504) | 14.4  (356) |
| Underweight (%) | 19.2  (623) | 17.4  (573) | 12.5  (468) | 8.4  (504) | 8.6  (356) |
| Wasting (%) | 9.4  (623) | 10.1  (573) | 9.4  (468) | 6.1  (504) | 6.6  (356) |
| Anaemia in children (%) | 87.4  (570) | 83.6  (543) | 81.5  (418) | 67.8  (457) | 61.2  (325) |
| ARI in children (%) | 6.2  (693) | 5.5  (610) | 4.6  (507) | 7.4  (528) | 2.9  (393) |
| Diarrhea in children | 25.3  (693) | 21.4  (610) | 21.5  (507) | 16.4  (528) | 10.2  (393) |
| BMI<18.5 (thin) (%) | 12.6  (668) | 14.3  (776) | 9  (875) | 5.2  (1031) | 4.9  (1030) |
| BMI 25-29.9 (overweight) (%) | 9.7  (668) | 12.6  (776) | 18.5  (875) | 29.5  (1031) | 27  (1030) |
| BMI>=30 (obese) (%) | 2.1  (668) | 3.2  (776) | 4.8  (875) | 11.7  (1031) | 19.8  (1030) |
| Anaemia in women (%) | 60.7  (754) | 63  (867) | 59.5  (959) | 57.9  (1086) | 53.9  (1081) |
| Treatment of diarrhea in children (%) | 39  (176) | 39  (131) | 42.9  (109) | 48  (86) | 41.2  (40) |
| Received all basic vaccines – children (%) | 74.5  (129) | 77.4  (124) | 75  (110) | 86.2  (110) | 84.3  (78) |
| Skilled attendance at birth (%) | 24.2  (744) | 50  (641) | 64.8  (549) | 81.7  (560) | 94.6  (415) |
| Delivery in health facility (%) | 23.5  (744) | 48.7  (641) | 62.1  (549) | 80.1  (560) | 92.8  (415) |
| Delivery in public sector health facility (%) | 22.1  (744) | 41.7  (641) | 53.5  (549) | 68.8  (560) | 71.5  (415) |
| Delivery in private sector health facility (%) | 1.4  (744) | 7  (641) | 8.6  (549) | 11.3  (560) | 21.2  (415) |
| Home delivery (%) | 75.7  (744) | 50.2  (641) | 36.5  (549) | 19.6  (560) | 6.6  (415) |
| Caesarean section (%) | 1.3  (744) | 5  (641) | 8.4  (549) | 9.1  (560) | 15  (415) |
| Use of modern contraceptive methods (%) | 11.6  (573) | 14.4  (577) | 15.8  (525) | 20  (600) | 20.6  (601) |
| Child slept under ITN (%) | 28.1  (1427) | 29.3  (1252) | 30.1  (1128) | 27.7  (1110) | 24.8  (874) |
| Pregnant woman slept under ITN (%) | 23  (65) | 22.4  (82) | 19.4  (68) | 14.4  (64) | 10.6  (74) |
| IPT during pregnancy | 31.2  (283) | 42.6  (261) | 50.3  (222) | 49.2  (243) | 49.8  (169) |

**Annex 1: Distribution of indicators by wealth quintile**
